# Observations on a Passage by Sir H. Davy

**Published:** 1812-11

**Authors:** James Woodham

**Affiliations:** Blackfriars


### *To the Editors of the Medical and Physical Journal.*

"Nam quod hypotheses spectat, eæ in philosophiâ experimentali locum habere non debent."

*Newton. Princip. Philos. c Comment. T. Le Seur et Fr. Jacquier.*

"The legitimate province of philosophy extends no farther than to conclusions resting on the solid basis of observation and experiment."

*Dug. Stewart.*

GENTLEMEN,

NATURE being a series of phenomena only, the business of philosophy I conceive is by observation and experiment to become acquainted with those phenomena, to arrange them, to ascertain their laws, and apply them to the improvement and well-being of man. Sound philosophy goes no farther. It rejects all hypothesis; it meddles not with causation; it confines itself to observation and experiment, and to induction from these alone.

I was led to these considerations from observing in the "Elements of Chemical Philosophy," just published by Sir Humphrey Davy, the following attempt to account for those appearances which present themselves in bodies that are heated. Rejecting the generally-received hypothesis, he observes, "It seems possible to account for all the phenomena of heat, if it be supposed, that, in solids, the particles are in a constant state of vibratory motion, the particles of the hottest bodies moving with the greatest velocity, and through the greatest space; that in fluids, and elastic fluids, besides the vibratory motion, which must be conceived greater in the last, the particles have a motion round their own axis with different velocities, the particles of elastic fluids moving with the greatest quickness; and that, in ethereal substances, the particles move round their own axis, and separate from each other, penetrating in right lines through space. Temperature may be conceived to depend upon the velocities of the vibrations; increase of capacity on the motion being performed in greater space; and the diminution of temperature during the conversion of solids into fluids or gasses, may be explained on the idea of the loss of vibratory motion in consequence of the revolution of particles round their axis, at the moment when the body becomes fluid or æriform, or from the loss of rapidity of vibration, in consequence of the motion of the particles through greater space."

Now I do not apprehend we can find in the writings of Epicurus or of Leibnitz, of Hartley or of Darwin, an hypothesis more extravagant. The atoms and monads of the two former, and the vibrations and sensorial motions of the two latter, are not, I conceive, more visionary than the vibratory particles particles revolving round their axis with increased or diminished velocities of Sir Humphrey. It appears too, that this hypothesis is not a new one, but merely an amplification of an old one, for, in an essay on "Heat, Light, &c." published by the late Dr. Beddoes in the "Contributions to Medical and Physical Knowledge," written by Sir Humphrey, then Mr., Davy, he says, "Heat is a sensation accompanying a peculiar motion, probably a vibration of the corpuscles of bodies tending to separate them;" so that, if Sir Humphrey's hypothesis is to be regarded as more correct, that of Mr. Davy must be acknowledged to be more simple, and equally I presume as near to truth.

*Blackfriars,*
*Sept.* 4, 1812.

I am, &c.
JAMES WOODHAM.

